# ROCK Inhibition Drives Resolution of Acute Inflammation by Enhancing Neutrophil Apoptosis

**DOI:** 10.3390/cells8090964

**Published:** 2019-08-23

**Authors:** Izabela Galvão, Rayssa M. Athayde, Denise A. Perez, Alesandra C. Reis, Luisa Rezende, Vivian Louise S. de Oliveira, Barbara M. Rezende, William A. Gonçalves, Lirlândia P. Sousa, Mauro M. Teixeira, Vanessa Pinho

**Affiliations:** 1 Departamento de Morfologia, Instituto de Ciências Biológicas, Universidade Federal de Minas Gerais, Belo Horizonte 31270-901, Brazil; 2 Departamento de Bioquímica e Imunologia, Instituto de Ciências Biológicas, Universidade Federal de Minas Gerais, Belo Horizonte 31270-901, Brazil; 3 Departamento de Enfermagem Básica, Escola de Enfermagem, Universidade Federal de Minas Gerais, Belo Horizonte 30130-100, Brazil; 4 Departamento de Análises Clínicas e Toxicológicas, Faculdade de Farmácia; Universidade Federal de Minas Gerais, Belo Horizonte 312701-901, Brazil

**Keywords:** acute inflammation, neutrophil, resolution of inflammation, ROCK, apoptosis, efferocytosis

## Abstract

Uncontrolled inflammation leads to tissue damage and it is central for the development of chronic inflammatory diseases and autoimmunity. An acute inflammatory response is finely regulated by the action of anti-inflammatory and pro-resolutive mediators, culminating in the resolution of inflammation and restoration of homeostasis. There are few studies investigating intracellular signaling pathways associated with the resolution of inflammation. Here, we investigate the role of Rho-associated kinase (ROCK), a serine/threonine kinase, in a model of self-resolving neutrophilic inflammatory. We show that ROCK activity, evaluated by P-MYPT-1 kinetics, was higher during the peak of lipopolysaccharide-induced neutrophil influx in the pleural cavity of mice. ROCK inhibition by treatment with Y-27632 decreased the accumulation of neutrophils in the pleural cavity and was associated with an increase in apoptotic events and efferocytosis, as evaluated by an in vivo assay. In a model of gout, treatment with Y-27632 reduced neutrophil accumulation, IL-1β levels and hypernociception in the joint. These were associated with reduced MYPT and IκBα phosphorylation levels and increased apoptosis. Finally, inhibition of ROCK activity also induced apoptosis in human neutrophils and destabilized cytoskeleton, extending the observed effects to human cells. Taken together, these data show that inhibition of the ROCK pathway might represent a potential therapeutic target for neutrophilic inflammatory diseases.

## 1. Introduction

A precise control of leukocyte accumulation in inflammatory sites is needed to avoid tissue damage and functional loss of inflamed tissue or organ [1,2,3]. There is a fine-tuned control of inflammatory response by stimulation of events associated with resolution of inflammation. It is now evident that resolution of acute inflammation is an active and programmed process highly dependent on the production of pro-resolutive mediators, apoptosis of leukocyte and macrophage reprogramming [4,5,6,7]. Studies have demonstrated that blockage of pro-survival signaling pathways, during inflammation, might induce apoptosis of leukocytes thereby activating the process of resolution. For example, PI3K and Akt inhibitors enhance apoptosis and promote granulocyte clearance, resulting in the resolution of acute inflammation [8,9,10]. Likewise, NF-κB pathway blockade has also been shown to be involved in inflammation resolution [10,11]. Defective apoptosis and clearance are associated with many inflammatory conditions, leading to chronic inflammatory response and autoimmunity [2,12]. Thus, targeting leukocyte survival might be a useful therapeutic strategy for inflammatory diseases. 

Rho-associated kinase (ROCK) was first described as a downstream effector of RhoA [13], a small GTPase associated with cytoskeleton. ROCK has been shown to display a broad range of cellular functions, including modulation of morphology, motility, adhesion, cellular recruitment, gene transcription, cytokinesis and cell survival [14,15,16]. ROCK is normally expressed in many tissue types such as blood, colon, peripheral nervous system and small intestine [16]. Expression and activation of ROCK has also been observed in endothelial cells and leukocytes during an inflammatory response [17,18]. Furthermore, the RhoA-ROCK pathway is relevant in phagocytosis of apoptotic cells, known as efferocytosis [19], an essential aspect of a successful resolution. Previous studies have demonstrated that inhibition of RhoA and/or ROCK promotes engulfment [20,21,22], showing a negative regulation of efferocytosis by this pathway [23,24]. The action of ROCK depends on the phosphorylation of a number of intracellular proteins, including the regulatory subunit of myosin light chain phosphatase—MYPT-1 [25,26]. 

ROCK has been implicated in the pathogenesis of various inflammatory diseases including cancer, diabetes and fibrogenic disease [27,28]. In this context, it has been demonstrated that ROCK inhibitors such as Y-27632 and Fasudil, exhibit anti-inflammatory effects during acute lung injury, rheumatoid arthritis and Crohn’s disease, by preventing NF-κB activation and inhibiting TNF-α and IL-1β production [29,30,31]. In addition, elevated expression of ROCK was detected in T cells isolated from systemic lupus erythematosus patients and pharmacological inhibition of ROCK by certain compounds, including Y-27632, decreased the production of IL-17 and IL-21, cytokines that contribute to the pathogenesis of autoimmune disorders [32]. However, the importance of ROCK in the survival of neutrophil and resolution of inflammation remains unknown. Here, we showed that ROCK activity was associated with the productive phase of acute inflammation and pharmacological inhibition of ROCK reduced the accumulation of neutrophils in the inflammatory site by inducing apoptosis and neutrophils clearance. Thus, ROCK inhibition could be an appropriate therapeutic strategy to induce resolution of established inflammatory response.

## 2. Materials and Methods 

### 2.1. Animals 

This study was carried out in accordance with the recommendations of the law 11.794 from the National Council for Control of Animals Experimentation—CONCEA, Brazil. The protocol was approved by the Animal Ethics Council–CEUA—at Universidade Federal de Minas Gerais (Protocol Number 336/2015 and 2/2015), Brazil. Male BALB/c and male C57Bl/6 (8–12 weeks) mice were obtained from the Center of Bioterism of Universidade Federal de Minas Gerais (UFMG) Brazil, and were housed under standard conditions and had free access to commercial food and water. 

### 2.2. Drugs, Reagents, and Antibodies

Y-27632 was purchased from Sigma-Aldrich (St. Louis, MO, USA) and Tocris Bioscience (Minneapolis, MN, USA). zVAD-fmk (Tocris Bioscience, Minneapolis, MN, USA). Rabbit anti-p-MYPT-1, anti-caspase-3, anti-cleaved caspase-3, p-IκBα and secondary anti-rabbit peroxidase-conjugate antibodies were from Cell Signaling Technology (Beverly, MA, USA). Secondary anti-mouse and anti-goat peroxidase-conjugate antibodies were purchased from Santa Cruz Biotechnology (Santa Cruz, CA, USA). Anti-β-actin and LPS (Lipopolysaccharide from *Escherichia coli* serotype O:111:B4) were from Sigma-Aldrich (St. Louis MO, USA). Phalloidin-Alexa Fluor 546 was purchased from Invitrogen (Carlsbad, CA, USA)

### 2.3. Leukocyte Migration into the Pleural Cavity Induced by LPS 

BALB/c mice received an intrapleural (i.pl.) administration of LPS (250 ng/cavity) or vehicle, as described previously [8]. Cells present in the pleural cavity were harvested at different times after administration of LPS by washing the cavity with 2 mL PBS (phosphate buffered saline) and total cell counts performed in a modified Neubauer chamber using Turk’s stain. Cell analysis was performed by flow cytometry, as described below. 

### 2.4. Treatment Protocols 

To assess the role of ROCK on the LPS-induced pleurisy, mice were treated locally (i.pl.) with Y-27632 (1 or 10 mg/kg) 4 h after LPS-challenged. Pleural wash was performed 4 h after treatment and cells were analyzed. To evaluate leukocyte apoptosis, zVAD-fmk (1 mg/kg), a broad-spectrum caspase inhibition, was given systemically (i.p.) 15 min before injecting Y-27632, which was dissolved in PBS. Control mice received drug vehicle only. 

To assess the role of ROCK on Gouty model, mice were treated systemically (i.p.) with Y-27632 (10 mg/kg) 12 h after uric acid challenged. Knee wash was performed 6 h or 4 h after treatment and cells were analyzed.

### 2.5. Flow Cytometry Analysis for Leukocyte Populations and Expression of P-MYPT1

**Figure 1:**
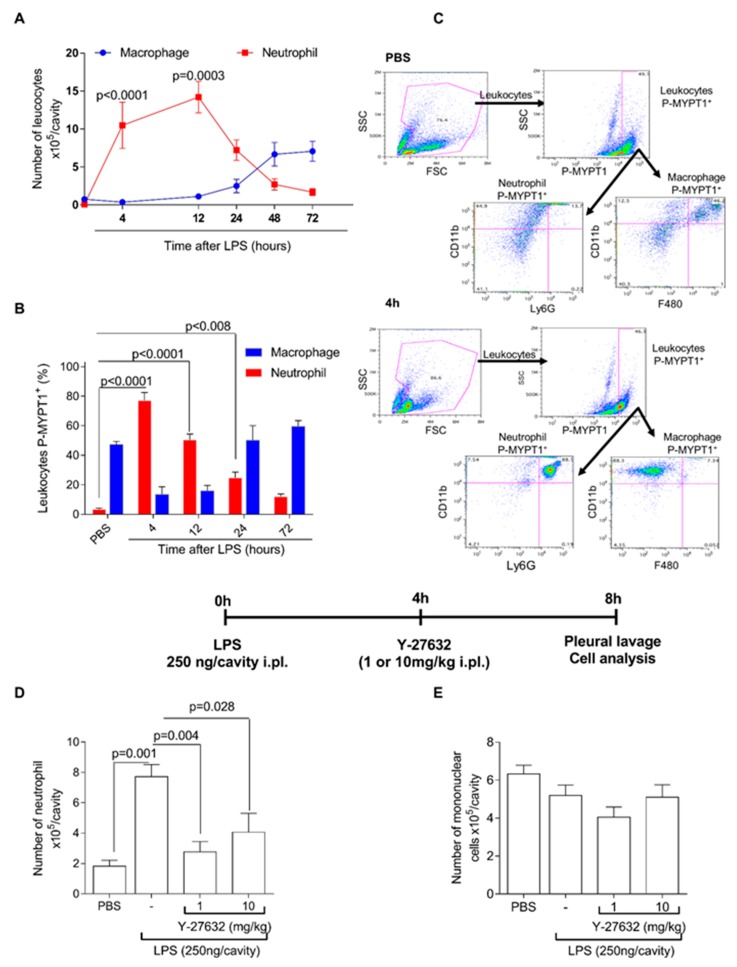
Time course of Rho-associated kinase (ROCK) activity during productive phase of lipopolysaccharide-induced pleurisy and the effects of ROCK inhibition. Mice were injected with phosphate buffered saline (PBS) or lipopolysaccharide (250 ng/cavity, i.pl.), and the number of leukocyte (neutrophils and macrophages, **A**) were evaluated at various times by flow cytometer. Data were collected with FACSCanto II flow cytometer and analyzed with FlowJo software. P-MYPT-1 expression was evaluated by flow cytometer at various times and represented as percentage of p-MYPT positive (**B**). Data were collected with BD Accuri™ C6 cytometer and analyzed with FlowJo software. Representative dot plots of gate strategy for the flow cytometric analysis (**C**). Mice were injected with PBS or LPS (250 ng/cavity, i.pl.), and 4 h later received two different dose of Y-27632 (1 or 10 mg/kg, i.pl.), a ROCK inhibitor in the peak of inflammation and four hours after treatment, cells were collected and processed to count number of neutrophils (**D**) and mononuclear cells (**E**). Results are expressed as the number of cells/cavity and are shown as the mean ± SEM of five mice in each group (ANOVA test followed by Holm-Sidak’s multiple comparison).

Mice received a local (i.pl.) LPS-injection and cells found present in the pleural cavity were harvested at different time points (4, 12, 24, 48 and 72 h after LPS-challenge). The populations of macrophages and neutrophils were analyzed by staining with fluorescent mAbs against F4/80 (PE—Biolegend, San Diego, CA, USA; PE-Cy7—eBioscience, San Diego, CA, USA), CD11b (PerCP-Cy5.5—BD Biosciences, San Jose, CA, USA; Alexa Fluor 488—BD Bioscience, San Jose, CA, USA), Ly6G (APC—BD Bioscience, San Jose, CA; V450—BD Bioscience, San Jose, CA, USA), P-MYPT1 (Cell Signaling Technology, Beverly, MA, USA), and anti-rabbit (Alexa 488—BD Biosciences, San Jose, CA, USA). After being stained for surface markers, cells were fixed by incubating with formaldehyde for 20 min. Then, the cells were washed and permeabilized with permeabilization buffer (Perm/Wash, BD Bioscience, San Diego, CA) for 30 min. After permeabilization, cells were stained with intracellular mAbs. Stained cells were acquired in a BD Accuri™ C6—Flow Cytometry or BD FCASCANTO II (both from BD Biosciences, San Jose, CA, USA) and analyzed using FlowJo software (Tree Star, Ashland, OR, USA). Gating strategy is illustrated in Figure 1. Neutrophil and macrophage were evaluated for P-MYPT1 expression. For this, cells selected in the side scatter/forward scatter gate were considered total leukocytes and were analyzed for P-MYPT1 expression. Then, P-MYPT1^+^ cells were separated into Ly6G^+^ (neutrophil) and F4/80^+^/CD11b^+^ (macrophage). P-MYPT1 labeling was performed at 1:50 dilution, and negative controls were cells stained only with fluorochrome-bound secondary antibodies anti-rabbit. 

### 2.6. Gouty Model

Uric acid crystals (MSU) were prepared using uric acid (Sigma Aldrich – St. Louis, MO, USA) as previously described [33]. C57Bl/6 mice were anesthetized (80:15 mg/kg ketamine:xylazine i.p., Syntec, São Paulo, Brazil) and received an injection into the tibiofemoral knee joint of 100 μg of MSU crystals. To evaluate inflammatory parameters, mice were euthanized and the knee cavity was washed with PBS/BSA 3% (2 × 5 μL) to harvest cells. Total cell counts were performed in a Neubauer chamber using Turk’s stain and differential counts were performed using morphologic criteria on a slide stained with May-Grunwald-Giensa. Periarticular tissue were collected from the knee to perform IL-1β measurement by ELISA assay.

### 2.7. Evaluation of Hypernociception

The mechanical hypernociception were evaluated as previously described in a blind manner [34] using an electronic pressuremeter (Insight instruments, Ribeirão Preto, SP, Brazil). The dorsiflexion-elicited withdrawal threshold was expressed in grams (g) and used to infer behavioral responses associated with experimental pain (hypernociception).

### 2.8. Calculation of Resolution Indices

We quantified the resolution indices as described [35,36]. Knee wash were collected from articular cavity at 12, 18, 24 and 36 h after MSU injection. The treatment with Y-27632 was performed in the peak of inflammation (12 h after MSU injection). The number of polymorphonuclear (PMN) and mononuclear cells were determined by total and differential leukocyte counting in a blind manner. The resolution of acute inflammation were defined in quantitative terms by the following resolution indices: (1) magnitude (ψmax and Tmax), ψmax (maximal PMN), Tmax (time point when PMN numbers reach maximum); (2) duration (T50), T50 (time point when PMN numbers reduce to 50% of maximum) and (3) resolution interval Ri (the interval between Tmax and T50, when 50% PMN are lost from the articular cavity).

### 2.9. Neutrophil Isolation and Culture 

Granulocytes were isolated from the peripheral blood of healthy donors as described elsewhere [37]. Briefly, blood was collected into ethylenediamine tetraacetic acid (EDTA) and was separated through a double-density gradient using Histopaque 10,771 and 11,191 (both Sigma-Aldrich; St. Louis MO, USA). After polymorphonuclear cell isolation and wash, contaminating erythrocytes were removed by hypotonic lysis. Neutrophil isolates were approximately 96% pure as confirmed by morphological appearance using light microscopy. Cells were ressuspended at 2 × 10^5^/mL in RPMI-1640 medium (Sigma-Aldrich; St. Louis MO, USA) and cultured for 2 h at 37 °C with 5% CO_2_ with Y-27632 (30 μM) and LPS (1 μg/mL). Incubation time was determined based on previous published works [38]. All subjects gave their informed consent for inclusion before they participated in the study. The study was conducted in accordance with the Declaration of Helsinki, and the protocol was approved by the Ethics Committee of Institutional Review Board Project number CAAE–0319.0.203.000-11.

### 2.10. Assessment of Leukocyte Apoptosis 

Apoptosis was assessed morphologically, as reported previously by our group [8,39]. Briefly, cells (5 × 10^4^) collected 8 h after LPS administration or 18 h after MSU injection were cyto-centrifuged, fixed, and stained with May-Grünwald-Giemsa and counted using oil immersion microscopy (×100 objective) to determine the proportion of cells with distinctive apoptotic morphology in a blind manner. This was assessed by recognizing cells presenting pyknosis—the nuclei are dense and compacted and have begun to break-up (a process named “karyorrhexis”) resulting in multiple spheres of dark-staining nuclear chromatin. At least 500 cells were counted/slide, and results are expressed as the mean ± SEM of percentage of cells with apoptotic morphology. Apoptosis was also evaluated by western blot, assessing the cleavage of caspase-3 and by flow cytometry. For in vivo experiments, cells were collected and surface-staining for 30 min with anti-LY6G-BV421 antibody (eBioscience) and then labelling with annexin-V APC and PI, and for in vitro experiments, isolated cells was labelled with annexin-V APC and PI as an index of loss of nuclear membrane integrity (PE Annexin V Apoptosis Detection Kit; BD PharmingenTM; San Jose, CA, USA).

### 2.11. Western Blotting 

Inflammatory cells harvested from the pleural cavity or human neutrophil after culture were washed with PBS and lysed on ice in lysis buffer (1% Triton X-100, 100 mM Tris/HCl, pH 8.0, 10% glycerol, 5 mM EDTA, 200 mM NaCl, 1 mM DTT, 1 mM PMSF, 2.5 μg/mL leupeptin, 5 μg/mL aprotinin, and 1 mM sodium orthovanadate). Protein amounts were quantified with the Bradford assay reagent from Bio-Rad (Hercules, CA, USA). Extracts (40–60 μg) were separated by electrophoresis on a denaturing, 8–15% polyacrylamide-SDS gel and electrotransferred to nitrocellulose membranes (Hybond ECL, GE Healthcare). Membranes were blocked with PBS containing 5% (*w/v*) nonfat dry milk and 0.1% Tween-20, washed three times with PBS containing 0.1% Tween-20, and then, incubated overnight (4 °C) with specific primary antibodies (P-MYPT-1, caspase-3, cleaved caspase-3, p-IκBα or β-actin) using a dilution of 1:1000 in PBS containing 5% (*w/v*) BSA and 0.1% Tween-20. After washing, membranes were incubated with appropriated HRP-conjugated secondary antibody (1:3000). Immunoreactive bands were visualized by using an ECL detection system, as described by the manufacturer (GE Healthcare, Piscataway, NJ, USA). The bands were quantified by densitometry, using the software Image J (version 1.51, National Institutes of Health, Bethesda, MD, USA, 2015). 

### 2.12. Efferocytosis Assay In Vivo

The in vivo efferocytosis assay was performed as previously described [37,40,41]. Human neutrophils were isolated as described in Neutrophil isolation and culture above. Apoptosis (>90%) was induced with staurosporine (10 µM) by culturing the neutrophils in complete RPMI 1640 for 1 h at 37 °C in a 5% CO_2_ atmosphere. A total of 3 × 10^6^ CFSE (10 μM)-labelled apoptotic PMNs were injected intraperitoneally into mice with 96 h of peritonitis elicited by 0.1 mg of zymosan. These mice were previously injected (30 min before apoptotic cells administration) with Y-27632 (10 mg/kg) or vehicle. Mice were euthanized after 30 min and peritoneal wash were performed in order to collect cells for analysis. Cells were surface-stained for 30 min with anti-F4/80-PECy7 antibody (eBioscience). Data were collected with BD Accuri™ C6—Flow Cytometry and analyzed with FlowJo software and efferocytosis was evaluated by F4/80^+^ and CFSE^+^.

### 2.13. Confocal Analysis

Cells were harvested from knee wash and cytocentrifuged (Cytospin; Shandon Lipshaw Inc., Pittsburgh, PA, USA) in cells coverslips. Next, cells were fixed in 4% paraformaldehyde for 20 min at room temperature. Cells were permeabilized for 10 min with triton-X-100 1% and then incubated with primary antibodies p-MYPT (Cell Signaling Technology, Danvers, MA, USA) overnight. The secondary antibody used was anti-rabbit Alexa-647 BD, USA) and nuclei were stained with DAPI (1:1000–BD). Finally, coverslips were prepared with fluoromount (Sigma Aldrich, USA) for confocal microscopy analysis. Images were obtained using the Ti microscope with laser confocal C2 equipped with three different lasers (excitation 405, 488 and 543 nm) and emission filters 450/50 nm (channel 1), 515/30 nm (channel 2) and 584/50 nm (channel 3). At least 500 cells per glass slide were counted and the fluorescence intensity was measured off-line using Volocity software 6.3 (Perkin-Elmer, Waltham, MA, USA). 

### 2.14. Phalloidin Staining

Human neutrophils were isolated, as described previously in this section, and recovered in glass coverslip through cytocentrifugation (Cytospin; Shandon Lipshaw Inc., Pittsburgh, PA, USA). Cells were then fixed with paraformaldehyde 4%, for 20 min at room temperature, and permeabilized with Tritonx-100 0.05% (Sigma-Aldrich) for 15 min. For labeling of polymerized actin fibers, we incubated the cells with Phalloidin-Alexa fluor 546 (1:50 in PBS for 20 min). After three washes, DNA were stained with DAPI, and the coverslips were mounted in glass slides for analysis in fluorescence microscope Zeiss Axio Vert.A1

### 2.15. Statistical Analysis 

All flow cytometry data were analyzed using FlowJo software (Treestar, Ashland, OR, USA). Human neutrophil experiments were conducted in triplicate using cells isolated from at least three different donors per experiment, and results are expressed as means ± SEM. Data were analyzed by one-way ANOVA and differences between groups were analyzed by post-test, Holm-Sidak’s multiple comparison post hoc test. A *p*-value < 0.05 was considered significant. Calculations were performed using the Prism 7.0 software program for Windows (GraphPad software, San Diego, CA, USA).

## 3. Results

### 3.1. ROCK Activity Is Associated with the Productive Phase of Lipopolysaccharide (LPS)-Induced Pleurisy and Its Inhibition Reduces Neutrophil Numbers in Pleural Cavity 

Initial experiments were performed in a mouse model of LPS-induced pleurisy, previously established by our group [8,39,42]. In this model, neutrophils accumulate in the pleural cavity of mice in a time-dependent manner, after LPS challenge. Using flow cytometry, we demonstrated that during the first 4 h after LPS challenge, neutrophil accumulation was significantly elevated and peaked at 4–12 h, gradually decreasing thereafter. The number of neutrophils decreased and reached basal levels after 48 h, a period that coincided with an influx of macrophages (Figure 1A) into the pleural cavity. 

We investigated whether ROCK activity was associated with neutrophil accumulation. Towards this, we evaluated the phosphorylation levels of MYPT-1 (P-MYPT-1), a well-established substrate of ROCK and a marker of ROCK activation [43]. As there are no neutrophils at baseline in naïve mice, we used as control, cells obtained from a cavity 24 h after injection of PBS, which induces very low number of neutrophils. Kinetic experiments showed that the number of P-MYPT-1^+^ cells paralleled the accumulation of neutrophils in the pleural cavity (Figure 1B). The level of expression of P-MYPT-1 by neutrophils was not altered between 4 and 24 h after injection of LPS (data not shown).

Thereafter, we evaluated the effects of ROCK inhibition on the course of LPS-induced pleurisy. To this end, we treated LPS-inflamed mice with Y-27632, a highly selective inhibitor of ROCK isoforms, 4 h after LPS challenge. After 4 h of treatment, cells were harvested and analyzed. As shown in Figure 1D, treatment with Y-27632 reduced the number of neutrophils in the pleural cavity in a dose dependent manner. However, Y-27632 did not affect the accumulation of mononuclear cells (Figure 1E). 

### 3.2. ROCK Inhibition Induces Caspase-Dependent Apoptosis of Neutrophils

**Figure 2:**
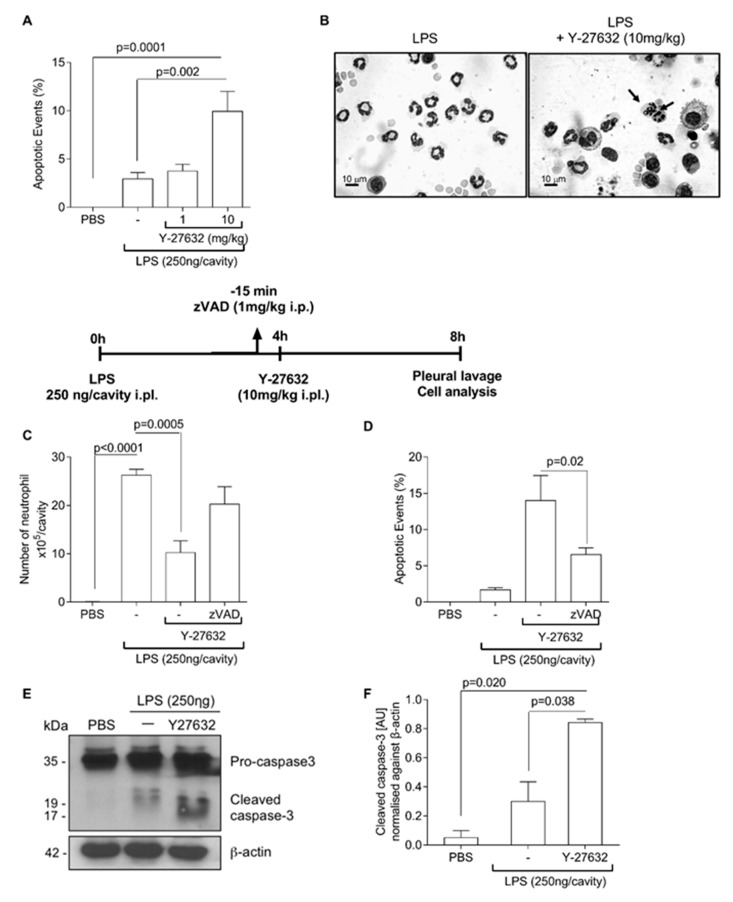
Effect of Y-27632 treatment on neutrophil apoptosis. Mice were injected with PBS or LPS (250 ng/cavity, i.pl.) and, 4 h later, received a local injection (i.pl.) of Y-27632 (1 or 10 mg/kg, i.pl.) or vehicle. Cells with distinctive apoptotic morphology were evaluated 4 h after drug treatment and are expressed as percent of neutrophils with distinctive apoptotic morphology (**A**) Representative figures of viable and apoptotic neutrophils (arrow) (**B**). Scale bar = 10 μm. Original magnifications, ×40. The pan-caspase inhibitor zVAD-fmk (1 mg/kg, i.p.) was given 15 min before ROCK inhibition (Y-27632 10 mg/kg, i.pl.). The number of neutrophils (**C**), cells with distinctive apoptotic morphology (**D**) and Western blot to detection of pro-caspase 3 and cleaved caspase-3 (**E**) were evaluated 4 h after drug treatment. Densitometry analysis of Western blot are shown (**F**). Results are expressed as the number of cells/cavity and are shown as the mean ± SEM of five mice in each group (ANOVA test followed by Holm-Sidak’s multiple comparison).

The reduction in the number of neutrophils in inflammatory sites might be associated with apoptosis and induction of resolution of inflammation [8,11,39]. We then examined the effects of Y-27632 on the apoptosis of neutrophils. The treatment with Y-27632 increased the number of apoptotic cells in the pleural cavity, as demonstrated by morphologic criteria (Figure 2A,B). In addition, the effects of ROCK inhibition on neutrophil survival were prevented by zVAD-fmk, a broad-spectrum caspase inhibitor (Figure 2C,D). Accordingly, mice treated with Y-27632 showed increased levels of caspase-3 cleavage (Figure 2E,F). Representative blots of two independent experiments are shown in Figure 2E. 

### 3.3. Y-27632 Enhances the Efferocytic Capacity of Macrophages 

**Figure 3:**
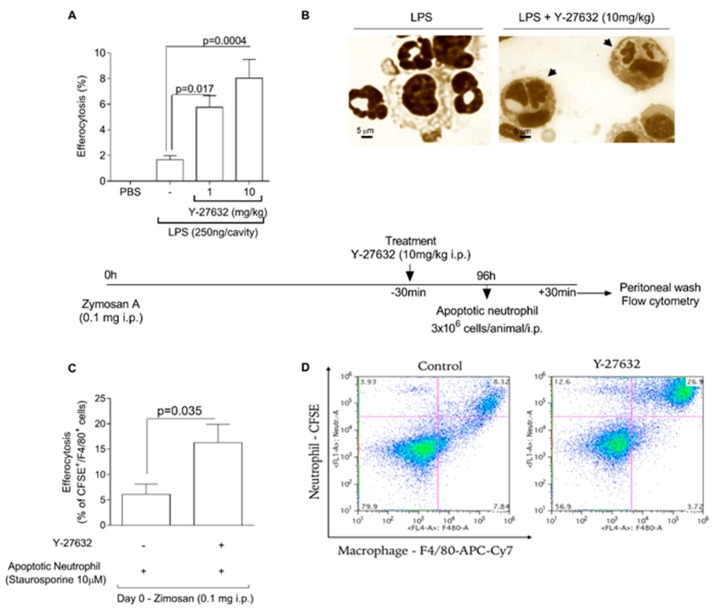
Y-27632 enhance efferocytic capacity of murine macrophage. Mice were injected with PBS or LPS (250 ng/cavity, i.pl.) and, 4 h later, received a local injection (i.pl.) of Y-27632 (1 or 10 mg/kg, i.pl.) or vehicle. Apoptotic cells inside macrophages were evaluated 4h after drug treatment. Results are expressed as percent of apoptotic neutrophils ingested by macrophages (**A**) Representative figure of viable neutrophil and macrophage in untreated mice and apoptotic neutrophil inside mononuclear cell (arrowhead) in animal treated with Y-27632 (**B**). Scale bar = 5μm. Original magnifications, ×40. Human neutrophils were isolated by histopaque gradient, and were incubated with staurosporine to induce apoptosis and fluorescently labeled with carboxyfluorescein succinimidyl ester (CSFE). Apoptotic neutrophils were injected (i.p) into mice bearing a 96-h peritonitis elicited by 0.1 mg of zymosan. ROCK inhibition (i.p.) was induced 30 min before apoptotic neutrophils injection. 30 min later cells were collected by peritoneal lavage and efferocytosis were evaluated by flow cytometer. Mice that received only apoptotic neutrophils were considered control group. Data were collected with BD Accuri™ C6—Flow Cytometry and analyzed with FlowJo software. Results are expressed as percent of double-positive cells for CFSE and F4/80 (**C**). Representative dotplots are shown (**D**). Results are shown as the mean ± SEM of five mice in each group (Student *t* test).

We investigated the capacity of Y-27632 to alter the phagocytosis of apoptotic neutrophils by mononuclear cells. For this, mice were injected with LPS and at the peak of inflammation (4 h) mice were treated with Y-27632. Four hours after treatment, cells were harvested from pleural cavity and cito-centrifuged to evaluate number of mononuclear cells that had engulfed apoptotic cells, a process called efferocytosis. Our results showed that Y-27632 treatment increased efferocytosis (Figure 3A,B). In addition, we also performed an in vivo efferocytosis assay, to assess the efferocytic capacity of macrophages after treatment with Y-27632. Towards this, human neutrophils undergoing apoptosis (after incubation with staurosporin - 10 μM) were fluorescently labelled with carboxyfluorescein succinimidyl ester (CFSE - 10μM) and injected intraperitoneally (i.p) into mice bearing a 96 h peritonitis induced by 0.1 mg of zymosan. Mice previously treated with Y-27632 presented a higher rate of efferocytosis when compared to vehicle-injected mice (Figure 3C,D). 

### 3.4. Inhibition of ROCK Reduced Neutrophil Accumulation, Hypernociception and IL-1β Expression Which Mediates Joint Inflammation

**Figure 4:**
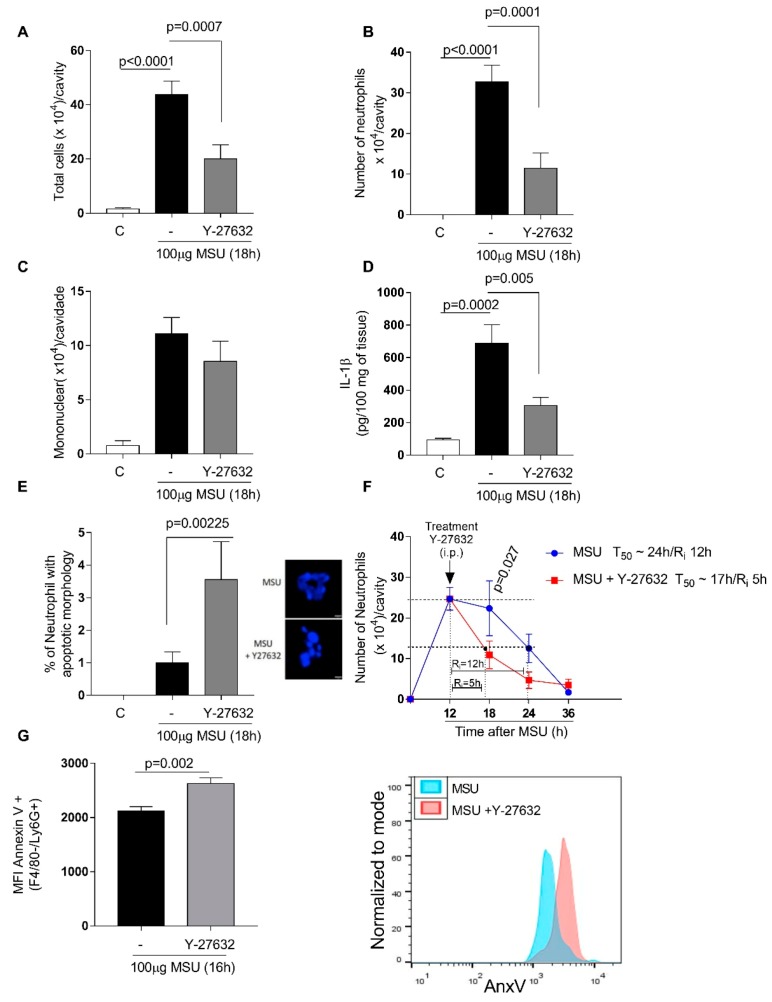
Effects of Y-27632 treatment on Monosodium Urate Crystals (MSU) - induced gout. C57BL/6J mice were injected with MSU crystals (100 μg) into the tibiofemoral joint and 12 h later received an injection of Y-27632 (10 mg/kg i.p.). Knee were washed 6 h after treatment to cell count total number cells (**A**), number of neutrophils (**B**) and number of mononuclear cells (**C**). Levels of IL-1β (**D**) were measured by ELISA and expressed in pg/100mg of periarticular tissue. Cells with distinctive apoptotic morphology were determined 6 h after treatment (**E**). Resolution indices were quantified (**F**). Of note, Tmax = 12 h, the time point when polymorphonuclear (PMN) numbers reach maximum; T_50_ Y-27632 ~17 h, the time point when PMN numbers reduce to 50% of maximum; and R_i_ Y-27632 ~5 h; R_i_, the time period when 50% PMNs are lost from the knee cavity. Apoptosis was biochemically 4 h after Y-27632 treatment and the median of fluorescence intensity (MFI) of annexin V was determined by flow cytometer (**G**). Data were collected with FACSCanto II flow cytometer and analyzed with FlowJo software. Data are shown as the mean ± SEM of five mice in each group (ANOVA test followed by Holm-Sidak’s multiple comparison).

To investigate the effect of ROCK inhibition in a disease model, mice were administered with an intraarticular injection of uric acid crystals (MSU 100 μg/cavity) into the tibiofemoral knee joint, to induce gout. The injection induced leukocyte recruitment to the joint, predominantly consistent by neutrophils. Treatment with Y-27632 during the peak of inflammation (12 h) reduced the number of leukocytes (Figure 4A), mostly neutrophils (Figure 4B) with no difference in the number of mononuclear cells (Figure 4C). The inhibition of ROCK activity reduced IL-1β levels in periarticular tissue (Figure 4D) and increased paw withdrawal threshold. The evaluation of hypernociception was performed using 5 mice/group. Statistical analysis was performed and a value of *p* < 0.05 was considered significant (Paw withdrawal threshold: MSU = 4.1 ± 0.1; MSU+Y-27632 = 5.1 ± 0.1; *p* = 0.003). Mice treated with Y-27632 exhibited increased proportion of cells with distinctive apoptotic morphology (Figure 4E) and shortened resolution indices (R_i_) (Figure 4F): R_i_ MSU ~12 h; R_i_ MSU+Y-27632 ~5 h; R_i_. The treatment also increased median fluorescence intensity of annexin-V in neutrophils harvested from knee (Figure 4G).

### 3.5. Treatment with Y-27632 Reduced MYPT1 and IκBα Phosphorylation in the Joint

**Figure 5:**
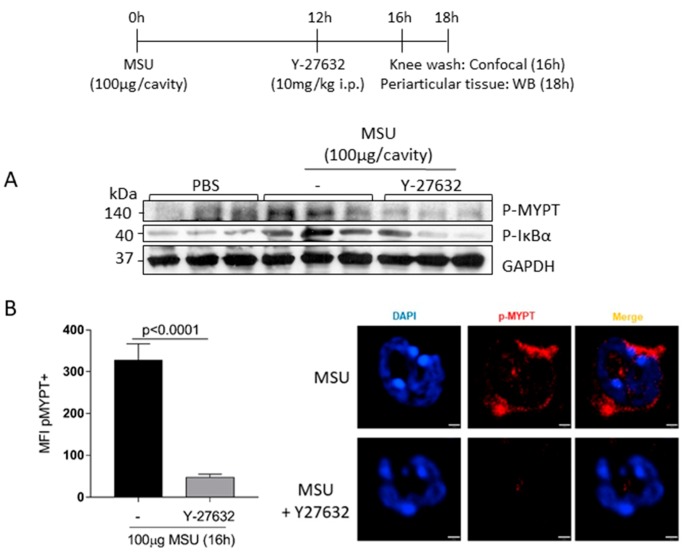
Effects of Y-27632 treatment on MYPT and IκBα phosphorylation. C57BL/6J mice were injected with MSU crystals (100 μg/cavity) into the tibiofemoral joint and 12 h later received an injection of Y-27632 (10 mg/kg i.p.). Periarticular tissue were collected 6 h after treatment to western blot analysis of p-MYPT and p-IκBα (**A**). Knee wash were collected 4 h after treatment and p-MYPT were evaluated by confocal analysis (**B**). The mean of fluorescence intensity (MFI) of pMYPT+ stained was measured off-line using Volocity software 6.3 (PerkinElmer). Representative figures are shown (**B**). Results are shown as the mean ± SEM of five mice in each group (Student *t* test).

To evaluate the mechanistic effect of Y-27632, western blotting analysis was performed to examine the phosphorylation levels of MYPT1 (P-MYPT), a substrate of ROCK and IκBα (p-IκBα), an important regulator of the pro-survival molecule NFκB in periarticular tissue from MSU-injected mice treated with or without Y-27632. We observed that, the treatment reduced the phosphorylation of MYPT1 and this was decrease was associated with reduced phosphorylation of IκBα, suggesting that inhibition of ROCK reduces activation of MYPT1 and subsequently down-regulates the regulator of the pro-survival molecule (Figure 5A). To confirm whether neutrophils were affected by the treatment with Y-27632, we performed confocal microscopy of cells harvested from the knee, and it were observed that treatment with Y-27632 abolished the phosphorylation of MYPT1 in neutrophils (Figure 5B).

### 3.6. ROCK Inhibition Promotes Apoptosis of Human Neutrophil 

**Figure 6:**
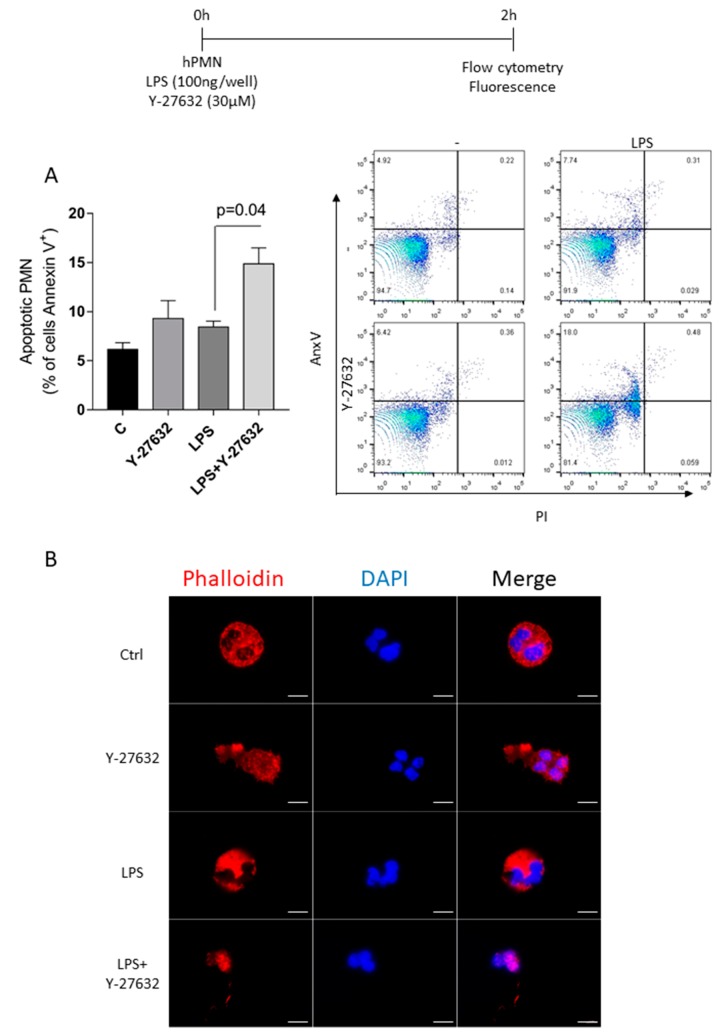
Effects of Y-27632 on human neutrophil apoptosis stimulated with LPS. Human peripheral blood samples were collected from healthy donors and isolated PMN were cultured for 2 h at 37 °C with 5% CO2 with the vehicle (C), treatment (Y-27632), stimulus (LPS) and both (Y-27632 + LPS). Apoptosis was determined by flow cytometer and are expressed as percentage of annexin V + cells (**A**). Data were collected with FACSCanto II flow cytometer and analyzed with FlowJo software. Representative dotplots are shown. Neutrophils were also stained with phalloidin and evaluated using a fluorescence microscope Zeiss Axio Vert A1 (**B**). Scale bar = 5 μm. Original magnifications, ×100. Results are shown as the mean ± SEM (ANOVA test followed by Holm-Sidak’s multiple comparison).

To provide a translational potential to these results, we investigated the effects of ROCK inhibition in human neutrophil. Human polymorphonuclear neutrophils (PMN) were cultured with Y-27632 (30μM) and LPS (1 μg/mL) for 2 h. Neutrophils stimulated with LPS and treated with Y-27632 presented a greater rate of apoptosis when compared to cells treated with only Y-27632, or stimulated only with LPS and non-treated cells (Figure 6A). Additionally, we also investigated whether 30 μM Y-27632 treatment affects neutrophil actin cytoskeleton through phalloidin staining. Control, non-treated neutrophils, showed a highly structured actin cytoskeleton, with actin fibers homogeneously distributed all over the cell. This was also observed in LPS treated cells even though the actin fibers in this condition appear to be thinner and more nucleated. Neutrophils only treated with Y-27632 showed less polymerized actin fibers that were mainly concentrated in some areas, which is typical of ruffle projections. Cells also lost their characteristic spherical shape and showed a more elongated and polarized morphology, a possible consequence of a more dynamic, less rigid cytoskeleton. Alterations of cell morphology were even more drastic in neutrophils treated with LPS and Y-27632, however in this condition, cells showed only few thin actin fibers close to the nucleus and in cell projections, indicating cell shrinkage and cytoskeleton collapse. 

## 4. Discussion

In this study we observed that: (1) ROCK activity was associated with the productive phase of acute inflammation induced by LPS; (2) the pharmacological inhibition of ROCK at the peak period of neutrophilic inflammation reduced the accumulation of neutrophils in the pleural cavity and (3) induced caspase-dependent apoptosis of neutrophils. Inhibiting ROCK activity (4) increased the efferocytic capacity of macrophage in vivo. In a model of gout, (5) inhibition of ROCK reduced neutrophil accumulation, IL-1β levels and hypernociception in the joint. These effects were associated with (6) reduction of MYPT and IκBα phosphorylation levels and increased apoptosis. Moreover, inhibition of ROCK activity also induced (7) apoptosis in human neutrophils and destabilization of the cytoskeleton. Therefore, we have shown in this study that ROCK activity might be involved in maintaining inflammatory responses, and modulation of this pathway could be a possible pro-resolutive strategy.

Our results showed increased ROCK expression that accompanied the neutrophil accumulation kinetics in the pleural cavity after administration of LPS. Other studies corroborate this finding, suggesting that optimal ROCK expression is required for maximum migration capacity of neutrophils [44]. Shi et al. have shown in a murine model of peritonitis that ROCK activation was required during neutrophil chemotaxis [44]. ROCK plays a role in leukocyte recruitment [18,45,46] and mediates the production of ROS and inflammatory cytokines [47]. Vemula et al. 2010 [48] demonstrated that ROCK-1 deficient mice had increased recruitment of leukocytes. These animals had no ROCK-1 expression but the expression of ROCK-2 was normal. As ROCK-1 deficient mice are not readily available and results hard to interpret, we chose not to use these animals. Therapeutic approaches aiming pharmacological inhibition of RhoA/ROCK pathway have proven effective. The use of fasudil induced dose-dependent suppression of neutrophil chemotaxis and also inhibited neutrophils-endothelial interaction induced by LPS through the reduction of expression of the adhesion molecule ICAM-1 [49]. Inhibition of ROCK exhibits anti-inflammatory properties, as demonstrated in a collagen-induced arthritis model in rats [30], in which treatment with two known ROCK inhibitors (fasudil and Y-27632) decreased synovial inflammation and suppressed the production of inflammatory cytokines. However, events associated with resolution of the inflammatory response have not been evaluated to the best of our knowledge. 

In the current study, we used Y-27632 as the major tool to investigate the role of ROCK inhibition in the context of inflammation resolution. It has been shown that this compound may affect, in part, the function of protein kinases A (PKA) and C (PKC). At the concentration used, the effects of this compound on PKA is not marked [50] and inhibition of PKA would likely be associated with opposite outcomes (ie. PKA activation favors resolution of inflammation) [8]. On the other hand, at concentrations used, there may be significant effects on PKC isoforms, especially PKCε, PKCη and PKCδ activity (IUPHAR drug data base). Evidence suggests that PKC isoenzymes are differentially involved in the regulation of apoptosis [51], however, there are no studies evaluating the effects of specific inhibitors in models of resolution of inflammation. Further studies are necessary to clarify whether an action on PKC would contribute to the effects of Y-27632 we observed in our system. 

Here, ROCK inhibition took place after neutrophil had migrated to the site of inflammation. The decrease in neutrophil accumulation in the pleural cavity induced by Y-27632 challenged with LPS was associated with the apoptosis of these cells. Increased apoptosis of neutrophils were also observed in a model of gout, suggesting that these effects were not stimulus dependent. The importance of granulocyte apoptosis for the resolution of acute inflammation has been demonstrated in several studies [8,9,39,52,53,54]. Apoptosis is considered a silent death by not releasing intracellular components that could act as pro-inflammatory mediators [55]. This type of death is initiated by the cleavage of caspase-3 and results in DNA fragmentation, degradation of cytoskeletal and nuclear proteins, externalization of phosphatidylserine, formation of apoptotic bodies, and finally uptake by phagocytic cells. All the biochemical modifications precede morphological features [56]. This sequence of biochemical events is in agreement with our findings [39,57,58,59] and results presented here). Furthermore, apoptotic granulocytes release factors that inhibit the recruitment of other effector neutrophils to the inflammatory site and stimulate the recruitment of non-inflammatory mononuclear cells [60]. Previous studies have shown that ROCK inhibition induces apoptosis in some cell types [14,15]. Y-27632 treatment induced apoptosis of hepatic stellate cells from rats, thyroid tumor cell lines, human endothelial cells, human epithelial cells and smooth muscle cells from rats [61,62,63,64,65]. Our results showed that Y-27632 treatment promotes neutrophil apoptosis during an inflammatory process, with activation of caspase-3. It is noteworthy that caspase blockade with zVAD-fmk prevented apoptosis and the consequent resolution of neutrophilic inflammation induced by ROCK inhibition. Apoptosis can proceed via either an extrinsic pathway on ligation of a death receptor or an intrinsic pathway in response to cellular stress. Both pathways are linked and molecules in one pathway can influence the other [66]. Thus, both pathways are responsible for the morphological and biochemical changes of apoptosis. However, there is no specific evidence for which pathways are involved in induction of neutrophil apoptosis in the resolution of inflammation context. Interestingly, induction of apoptosis appeared to be neutrophil specific as we did not find significant difference in the number of mononuclear cells after treatment with Y-27632 as compared to the untreated group. This is a relevant fact, since mononuclear cells are very important during the resolution process by secreting anti-inflammatory and/or pro-resolutive mediators and by phagocytosing apoptotic cells [6,67].

It is worth considering that ROCK increase vascular permeability by modulation actin polymerization in endothelial cells [68]. However, the relationship between vascular permeability and neutrophil influx is not direct. Although these phenomena can occur in parallel, they are not necessarily related [69]. It is also unknown whether any change in vascular permeability will affect the resolution of inflammation and neutrophil efflux. Therefore, an effect of ROCK inhibition on endothelial cells and changes vascular permeability may affect resolution of inflammation, and this possibility deserves further investigation in the future. 

The process of cell death is strongly connected to the removal of apoptotic bodies and cell debris [70]. If left unremoved, apoptotic cells may progress to late apoptosis and consequent necrosis, generating a pro-inflammatory profile. A rapid and efficient apoptotic leukocyte efferocytosis is a crucial phenomenon for the safe removal of these cells, preventing cell lysis and release of pro-inflammatory components to surrounding tissue [71,72]. The regulation of two distinct types of phagocytosis by different Rho family GTPases has been described [73], showing an opposite effect of these GTPases on the phagocytosis of apoptotic cells by macrophages. Simultaneously, Rac1 increases the rate of efferocytosis, RhoA, one of the activators of ROCK, inhibits this process [24]. Moreover, between the RhoA effector molecules, ROCK seems to be mainly responsible for this inhibitory effect [23]. Interestingly, Boe et al. (2010) have shown that acute alcohol exposure prevents efferocytosis in the lung of mice and treatment with Y-27632 reversed the effects of alcohol on efferocytosis, suggesting that these effects appear to be mediated, at least in part, by ROCK activation independent of RhoA [74]. These data show that ROCK could be the main effector in the inhibition of efferocytosis, even without the activation of RhoA. Other studies have also shown that ROCK inhibition increases efferocytosis during lung inflammation and chronic obstructive pulmonary disease (COPD) [20,75]. Our data corroborates with the literature and demonstrates that ROCK inhibition increased the rate of peritoneal macrophage efferocytosis in vivo. This was a relevant finding because, as mentioned earlier, induction of granulocyte apoptosis alone is not adequate for appropriate resolution, and careful clearance of these cells from the site of inflammation is essential. Thus, Y-27632 seems to meet some of the requirements necessary for a complete and effective resolution. Our results are the first to show the pro-resolutive role of pharmacological inhibition of ROCK during acute neutrophilic inflammatory response modifying the course of an established inflammation. These data contribute to a better understanding of ROCK signaling pathway suggesting a possible target for strategies that aim to accelerate and/or activate resolution programs. 

Interestingly, ROCK inhibition also reduced neutrophils accumulation, IL-1β levels and mechanical hypernociception in the mouse model of gout, which are essential inflammatory parameters in gout model [33], suggesting that inhibition of ROCK ameliorates inflammatory parameters by inducing apoptosis in neutrophil in the joint cavity. Treatment with Y-27632 impaired the phosphorylation of MYPT1, a ROCK substrate, in neutrophils recovered from the joint cavity. Moreover, the reduction of MYPT-1 phosphorylation was associated with a reduced IκBα activity, an important regulator of NFκB. It is well known that ROCK activates NFκB by different mechanisms [76,77,78]. Rho kinase inhibition prevents nuclear factor kappa B activation and I-kappa B phosphorylation and degradation [31]. Prevention of NFκB activation and reduced levels of IκBα phosphorylation was associated with resolution of inflammation [35,59,79,80]. We used different mouse backgrounds because the models of pleurisy induced by LPS and gout were established in the Balb/C and C57Bl/6, respectively, hence their use. In both systems, there are much data suggesting that both systems share the same pathway for neutrophil recruitment and survival [35,59,79,80]. Thus, the requirement of same pathway in different systems to neutrophil survival highlights the importance of this pathway to inflammation resolution. 

To provide a translational potential to these results, we investigate the role of ROCK inhibition in human cells. Our results indicate that ROCK inhibition also induces apoptosis of human neutrophils stimulated with LPS, by induction of phosphatidylserine (PtdSer) exposure. This effect was associated with complete mischaracterization of actin cytoskeleton. ROCK has been implicated in several RhoA-mediated responses such regulation of cytoskeletal dynamics, actin stress fibre formation and myosin L chain (MLC) phosphorylation. ROCK directly phosphorylates MLC or a regulatory subunit of myosin light chain phosphatase—MYPT1, that consequently inhibits MLCP activity and MLC dephosphorylation [16,26]. Thus, ROCK inhibition induces loss of stress fibers and reorganization of actin microfilaments to form short fibres that bundle together and increase the tensile strength of the cell and as a consequence of retraction, cells undergo apoptosis round [15]. These observations suggest that changes in the cytoskeleton not only impair the migration capacity of neutrophils but may be associated with increase of apoptosis during inflammation. However, the direct association of cytoskeleton and leukocyte death should be better investigated in the context of resolution of inflammatory response.

In conclusion, the present study showed that ROCK inhibitor switches off the activation of MYPT1 and NF-κB pathway and induces the resolution of acute inflammatory diseases. Thus, ROCK inhibitor may be a potential therapeutic strategy in inflammatory response where neutrophil accumulation is mandatory.

## References

[1] Medzhitov R. (2010). Inflammation 2010: New Adventures of an Old Flame. Cell.

[2] Nathan C. (2002). Points of Control in Inflammation. Nature.

[3] Medzhitov R. (2008). Origin and Physiological Roles of Inflammation. Nature.

[4] Ortega-Gómez A., Perretti M., Soehnlein O. (2013). Resolution of Inflammation: An Integrated View. Embo. Mol. Med..

[5] Serhan C.N. (2010). Novel Lipid Mediators and Resolution Mechanisms in Acute Inflammation: To Resolve or Not?. Am. J. Pathol..

[6] Serhan C.N., Brain S.D., Buckley C.D., Gilroy D.W., Haslett C., O’Neill L.A.J., Perretti M., Rossi A.G., Wallace J.L. (2007). Resolution of Inflammation: State of the Art, Definitions and Terms. FASEB J..

[7] Serhan C.N., Chiang N., Dalli J. (2015). The Resolution Code of Acute Inflammation: Novel pro-Resolving Lipid Mediators in Resolution. Semin. Immunol..

[8] Sousa L.P., Lopes F., Silva D.M., Tavares L.P., Vieira A.T., Rezende B.M., Carmo A.F., Russo R.C., Garcia C.C., Bonjardim C.A. (2010). PDE4 Inhibition Drives Resolution of Neutrophilic Inflammation by Inducing Apoptosis in a PKA-PI3K/Akt-Dependent and NF-KappaB-Independent Manner. J. Leukoc. Biol..

[9] Pinho V., Souza D.G., Barsante M.M., Hamer F.P., De Freitas M.S., Rossi A.G., Teixeira M.M. (2005). Phosphoinositide-3 Kinases Critically Regulate the Recruitment and Survival of Eosinophils in Vivo: Importance for the Resolution of Allergic Inflammation. J. Leukoc. Biol..

[10] Sousa L.P., Carmo A.F., Rezende B.M., Lopes F., Silva D.M., Alessandri A.L., Bonjardim C.A., Rossi A.G., Teixeira M.M., Pinho V. (2009). Cyclic AMP Enhances Resolution of Allergic Pleurisy by Promoting Inflammatory Cell Apoptosis via Inhibition of PI3K/Akt and NF-ΚB. Biochem. Pharmacol..

[11] Lopes F., Coelho F.M., Costa V.V., Vieira É.L.M., Sousa L.P., Silva T.A., Vieira L.Q., Teixeira M.M., Pinho V. (2011). Resolution of Neutrophilic Inflammation by H_2_O_2_ in Antigen-Induced Arthritis. Arthritis Rheum..

[12] Lawrence T., Gilroy D.W. (2007). Chronic Inflammation: A Failure of Resolution?. Int. J. Exp. Pathol..

[13] Matsui T., Amano M., Yamamoto T., Chihara K., Nakafuku M., Ito M., Nakano T., Okawa K., Iwamatsu A., Kaibuchi K. (1996). Rho-Associated Kinase, a Novel Serine/Threonine Kinase, as a Putative Target for Small GTP Binding Protein Rho. EMBO J..

[14] Shi J., Wei L. (2007). Rho Kinase in the Regulation of Cell Death and Survival. Arch. Immunol. Ther. Exp..

[15] Street C.A., Bryan B.A. (2011). Rho Kinase Proteins—Pleiotropic Modulators of Cell Survival and Apoptosis. Anticancer. Res..

[16] Julian L., Olson M.F. (2014). Rho-Associated Coiled-Coil Containing Kinases (ROCK): Structure, Regulation, and Functions. Small GTPases.

[17] Mong P.Y., Wang Q. (2009). Activation of Rho Kinase Isoforms in Lung Endothelial Cells during Inflammation. J. Immunol..

[18] Kikuchi Y., Yasuda S., Aizawa K., Tsuburaya R., Ito Y., Takeda M., Nakayama M., Ito K., Takahashi J., Shimokawa H. (2011). Enhanced Rho-Kinase Activity in Circulating Neutrophils of Patients with Vasospastic Angina: A Possible Biomarker for Diagnosis and Disease Activity Assessment. J. Am. Coll. Cardiol..

[19] deCathelineau A.M., Henson P.M. (2003). The Final Step in Programmed Cell Death: Phagocytes Carry Apoptotic Cells to the Grave. Essays Biochem..

[20] Bewley M.A., Belchamber K.B., Chana K.K., Budd R.C., Donaldson G., Wedzicha J.A., Brightling C.E., Kilty I., Donnelly L.E., Barnes P.J. (2016). Differential Effects of P38, MAPK, PI3K or Rho Kinase Inhibitors on Bacterial Phagocytosis and Efferocytosis by Macrophages in COPD. PLoS ONE.

[21] Richens T.R., Linderman D.J., Horstmann S.A., Lambert C., Xiao Y.Q., Keith R.L., Boé D.M., Morimoto K., Bowler R.P., Day B.J. (2009). Cigarette Smoke Impairs Clearance of Apoptotic Cells through Oxidant-Dependent Activation of RhoA. Am. J. Respir. Crit. Care Med..

[22] Vandivier R.W., Richens T.R., Horstmann S.A., DeCathelineau A.M., Ghosh M., Reynolds S.D., Xiao Y.-Q., Riches D.W., Plumb J., Vachon E. (2009). Dysfunctional Cystic Fibrosis Transmembrane Conductance Regulator Inhibits Phagocytosis of Apoptotic Cells with Proinflammatory Consequences. Am. J. Physiol. Lung Cell. Mol. Physiol..

[23] Tosello-Trampont A.C., Nakada-Tsukui K., Ravichandran K.S. (2003). Engulfment of Apoptotic Cells Is Negatively Regulated by Rho-Mediated Signaling. J. Biol. Chem..

[24] Nakaya M., Tanaka M., Okabe Y., Hanayama R., Nagata S. (2006). Opposite Effects of Rho Family GTPases on Engulfment of Apoptotic Cells by Macrophages. J. Biol. Chem..

[25] Amano M., Ito M., Kimura K., Fukata Y., Chihara K., Nakano T., Matsuura Y., Kaibuchi K. (1996). Phosphorylation and Activation of Myosin by Rho-Associated Kinase (Rho-Kinase). J. Biol. Chem..

[26] Kimura K., Ito M., Amano M., Chihara K., Fukata Y., Nakafuku M., Yamamori B., Feng J., Nakano T., Okawa K. (1996). Regulation of Myosin Phosphatase by Rho and Rho-Associated Kinase (Rho-Kinase). Science (N. Y. NY).

[27] Guan R., Xu X., Chen M., Hu H., Ge H., Wen S., Zhou S., Pi R. (2013). Advances in the Studies of Roles of Rho/Rho-Kinase in Diseases and the Development of Its Inhibitors. Eur. J. Med. Chem..

[28] Bei Y., Hua-Huy T., Nicco C., Duong-Quy S., Le-Dong N.N., Tiev K.P., Chereau C., Batteux F., Dinh-Xuan A.T. (2016). RhoA/Rho-Kinase Activation Promotes Lung Fibrosis in an Animal Model of Systemic Sclerosis. Exp. Lung Res..

[29] Tasaka S., Koh H., Yamada W., Shimizu M., Ogawa Y., Hasegawa N., Yamaguchi K., Ishii Y., Richer S.E., Doerschuk C.M. (2005). Attenuation of Endotoxin-Induced Acute Lung Injury by the Rho Associated Kinase Inhibitor, Y-27632. Am. J. Respir. Cell Mol. Biol..

[30] He Y., Xu H., Liang L., Zhan Z., Yang X., Yu X., Ye Y., Sun L. (2008). Antiinflammatory Effect of Rho Kinase Blockade via Inhibition of NF-KappaB Activation in Rheumatoid Arthritis. Arthritis Rheum..

[31] Segain J.P., De la Blétière D.R., Sauzeau V., Bourreille A., Hilaret G., Cario-Toumaniantz C., Pacaud P., Galmiche J.P., Loirand G. (2003). Rho Kinase Blockade Prevents Inflammation via Nuclear Factor ΚB Inhibition: Evidence in Crohn’s Disease and Experimental Colitis. Gastroenterology.

[32] Rozo C., Chinenov Y., Maharaj R.K., Gupta S., Leuenberger L., Kirou K.A., Bykerk V.P., Goodman S.M., Salmon J.E., Pernis A.B. (2017). Targeting the RhoA-ROCK Pathway to Reverse T-Cell Dysfunction in SLE. Ann. Rheum. Dis..

[33] Amaral F.A., Costa V.V., Tavares L.D., Sachs D., Coelho F.M., Fagundes C.T., Soriani F.M., Silveira T.N., Cunha L.D., Zamboni D.S. (2012). NLRP3 Inflammasome-Mediated Neutrophil Recruitment and Hypernociception Depend on Leukotriene B4 in a Murine Model of Gout. Arthritis Rheum..

[34] Amaral F.A., Bastos L.F.S., Oliveira T.H.C., Dias A.C.F., Oliveira V.L.S., Tavares L.D., Costa V.V., Galvão I., Soriani F.M., Szymkowski D.E. (2016). Transmembrane TNF-α Is Sufficient for Articular Inflammation and Hypernociception in a Mouse Model of Gout: Innate Immunity. Eur. J. Immunol..

[35] Galvão I., Queiroz-Junior C.M., de Oliveira V.L.S., Pinho V., Hirsch E., Teixeira M.M. (2019). The Inhibition of Phosphoinositide-3 Kinases Induce Resolution of Inflammation in a Gout Model. Front. Pharmacol..

[36] Galvão I., Vago J.P., Barroso L.C., Tavares L.P., Queiroz-Junior C.M., Costa V.V., Carneiro F.S., Ferreira T.P., Silva P.M.R., Amaral F.A. (2017). Annexin A1 Promotes Timely Resolution of Inflammation in Murine Gout. Eur. J. Immunol..

[37] Montero-Melendez T., Patel H.B., Seed M., Nielsen S., Jonassen T.E.N., Perretti M. (2011). The Melanocortin Agonist AP214 Exerts Anti-Inflammatory and Proresolving Properties. Am. J. Pathol..

[38] Lucas C.D., Allen K.C., Dorward D.A., Hoodless L.J., Melrose L.A., Marwick J.A., Tucker C.S., Haslett C., Duffin R., Rossi A.G. (2013). Flavones Induce Neutrophil Apoptosis by Down-Regulation of Mcl-1 via a Proteasomal-Dependent Pathway. FASEB J..

[39] Vago J.P., Nogueira C.R.C., Tavares L.P., Soriani F.M., Lopes F., Russo R.C., Pinho V., Teixeira M.M., Sousa L.P. (2012). Annexin A1 Modulates Natural and Glucocorticoid-Induced Resolution of Inflammation by Enhancing Neutrophil Apoptosis. J. Leukoc. Biol..

[40] Vago J.P., Sugimoto M.A., Lima K.M., Negreiros-Lima G.L., Baik N., Teixeira M.M., Perretti M., Parmer R.J., Miles L.A., Sousa L.P. (2019). Plasminogen and the Plasminogen Receptor, Plg-RKT, Regulate Macrophage Phenotypic, and Functional Changes. Front. Immunol..

[41] Newson J., Stables M., Karra E., Arce-Vargas F., Quezada S., Motwani M., Mack M., Yona S., Audzevich T., Gilroy D.W. (2014). Resolution of Acute Inflammation Bridges the Gap between Innate and Adaptive Immunity. Blood.

[42] Vago J.P., Tavares L.P., Garcia C.C., Lima K.M., Perucci L.O., Vieira É.L., Nogueira C.R.C., Soriani F.M., Martins J.O., Silva P.M.R. (2015). The Role and Effects of Glucocorticoid-Induced Leucine Zipper in the Context of Inflammation Resolution. J. Immunol. (Baltim. Md. 1950).

[43] Riento K., Ridley A.J. (2003). Rocks: Multifunctional Kinases in Cell Behaviour. Nat. Rev. Mol. Cell Biol..

[44] Shi Y., Zhang J., Mullin M., Dong B., Alberts A.S., Siminovitch K.A. (2009). The MDial Formin Is Required for Neutrophil Polarization, Migration, and Activation of the LARG/RhoA/ROCK Signaling Axis during Chemotaxis. J. Immunol..

[45] Palani K., Rahman M., Hasan Z., Zhang S., Qi Z., Jeppsson B., Thorlacius H. (2012). Rho-Kinase Regulates Adhesive and Mechanical Mechanisms of Pulmonary Recruitment of Neutrophils in Abdominal Sepsis. Eur. J. Pharmacol..

[46] Santen S., Wang Y., Laschke M.W., Menger M.D., Jeppsson B., Thorlacius H. (2010). Rho-Kinase Signaling Regulates CXC Chemokine Formation and Leukocyte Recruitment in Colonic Ischemia-Reperfusion. Int. J. Colorectal Dis..

[47] Shiotani S., Shimada M., Suehiro T., Soejima Y., Yosizumi T., Shimokawa H., Maehara Y. (2004). Involvement of Rho-Kinase in Cold Ischemia-Reperfusion Injury after Liver Transplantation in Rats. Transplantation.

[48] Vemula S., Shi J., Hanneman P., Wei L., Kapur R. (2010). ROCK1 Functions as a Suppressor of Inflammatory Cell Migration by Regulating PTEN Phosphorylation and Stability. Blood.

[49] Wang J., Xu J., Zhao X., Xie W., Wang H., Kong H. (2018). Fasudil Inhibits Neutrophil-Endothelial Cell Interactions by Regulating the Expressions of GRP78 and BMPR2. Exp. Cell Res..

[50] Tamura M., Nakao H., Yoshizaki H., Shiratsuchi M., Shigyo H., Yamada H., Ozawa T., Totsuka J., Hidaka H. (2005). Development of Specific Rho-Kinase Inhibitors and Their Clinical Application. Biochim. Biophys. Acta (BBA)—Proteins Proteom..

[51] Webb P.R., Wang K.Q., Scheel-Toellner D., Pongracz J., Salmon M., Lord J.M. (2000). Regulation of Neutrophil Apoptosis: A Role for Protein Kinase C and Phosphatidylinositol-3-Kinase. Apoptosis.

[52] Reis A.C., Alessandri A.L., Athayde R.M., Perez D.A., Vago J.P., Ávila T.V., Ferreira T.P.T., de Arantes A.C.S., de Sá Coutinho D., Rachid M.A. (2015). Induction of Eosinophil Apoptosis by Hydrogen Peroxide Promotes the Resolution of Allergic Inflammation. Cell Death Dis..

[53] Lucas C.D., Dorward D.A., Sharma S., Rennie J., Felton J.M., Alessandri A.L., Duffin R., Schwarze J., Haslett C., Rossi A.G. (2015). Wogonin Induces Eosinophil Apoptosis and Attenuates Allergic Airway Inflammation. Am. J. Respir Crit. Care Med..

[54] Leitch A.E., Lucas C.D., Marwick J.A., Duffin R., Haslett C., Rossi A.G. (2012). Cyclin-Dependent Kinases 7 and 9 Specifically Regulate Neutrophil Transcription and Their Inhibition Drives Apoptosis to Promote Resolution of Inflammation. Cell Death Differ..

[55] Hallett J.M., Leitch A.E., Riley N.A., Duffin R., Haslett C., Rossi A.G. (2008). Novel Pharmacological Strategies for Driving Inflammatory Cell Apoptosis and Enhancing the Resolution of Inflammation. Trends Pharmacol. Sci..

[56] Elmore S. (2007). Apoptosis: A Review of Programmed Cell Death. Toxicol. Pathol..

[57] Savill J. (1997). Apoptosis in Resolution of Inflammation. J. Leukoc. Biol..

[58] Taylor E.L., Rossi A.G., Dransfield I., Hart S.P., Quinn M.T., DeLeo F.R., Bokoch G.M. (2007). Analysis of Neutrophil Apoptosis. Neutrophil Methods and Protocols.

[59] Vago J.P., Tavares L.P., Sugimoto M.A., Lima G.L.N., Galvão I., de Caux T.R., Lima K.M., Ribeiro A.L.C., Carneiro F.S., Nunes F.F.C. (2016). Proresolving Actions of Synthetic and Natural Protease Inhibitors Are Mediated by Annexin A1. J. Immunol..

[60] Soehnlein O., Lindbom L. (2010). Phagocyte Partnership during the Onset and Resolution of Inflammation. Nat. Rev. Immunol..

[61] Ikeda H., Nagashima K., Yanase M., Tomiya T., Arai M., Inoue Y., Tejima K., Nishikawa T., Omata M., Kimura S. (2003). Involvement of Rho/Rho Kinase Pathway in Regulation of Apoptosis in Rat Hepatic Stellate Cells. Am. J. Physiol. Gastrointest. Liver Physiol..

[62] Zhong W.-B., Wang C.-Y., Chang T.-C., Lee W.-S. (2003). Lovastatin Induces Apoptosis of Anaplastic Thyroid Cancer Cells via Inhibition of Protein Geranylgeranylation and de Novo Protein Synthesis. Endocrinology.

[63] Li X., Liu L., Tupper J.C., Bannerman D.D., Winn R.K., Sebti S.M., Hamilton A.D., Harlan J.M. (2002). Inhibition of Protein Geranylgeranylation and RhoA/RhoA Kinase Pathway Induces Apoptosis in Human Endothelial Cells. J. Biol. Chem..

[64] Moore M., Marroquin B.A., Gugliotta W., Tse R., White S.R. (2004). Rho Kinase Inhibition Initiates Apoptosis in Human Airway Epithelial Cells. Am. J. Respir. Cell Mol. Biol..

[65] Shibata R., Kai H., Seki Y., Kusaba K., Takemiya K., Koga M., Jalalidin A., Tokuda K., Tahara N., Niiyama H. (2003). Rho-Kinase Inhibition Reduces Neointima Formation after Vascular Injury by Enhancing Bax Expression and Apoptosis. J. Cardiovasc. Pharmacol..

[66] Igney F.H., Krammer P.H. (2002). Death and Anti-Death: Tumour Resistance to Apoptosis. Nat. Rev. Cancer.

[67] Gilroy D.W., Lawrence T., Perretti M., Rossi A.G. (2004). Inflammatory Resolution: New Opportunities for Drug Discovery. Nat. Rev. Drug Discov..

[68] DiStasi M.R., Ley K. (2009). Opening the Flood-Gates: How Neutrophil-Endothelial Interactions Regulate Permeability. Trends Immunol..

[69] Wedmore C.V., Williams T.J. (1981). Control of Vascular Permeability by Polymorphonuclear Leukocytes in Inflammation. Nature.

[70] Gregory C.D., Pound J.D. (2011). Cell Death in the Neighbourhood: Direct Microenvironmental Effects of Apoptosis in Normal and Neoplastic Tissues. J. Pathol..

[71] Hart S.P., Dransfield I., Rossi A.G. (2008). Phagocytosis of Apoptotic Cells. Methods.

[72] Savill J., Dransfield I., Gregory C., Haslett C. (2002). A Blast from the Past: Clearance of Apoptotic Cells Regulates Immune Responses. Nat. Rev. Immunol..

[73] Caron E., Hall A. (1998). Identification of Two Distinct Mechanisms of Phagocytosis Controlled by Different Rho GTPases. Science (N. Y. NY).

[74] Boé D.M., Richens T.R., Horstmann S.A., Burnham E.L., Janssen W.J., Henson P.M., Moss M., Vandivier R.W. (2010). Acute and Chronic Alcohol Exposure Impair the Phagocytosis of Apoptotic Cells and Enhance the Pulmonary Inflammatory Response. Alcohol. Clin. Exp. Res..

[75] Moon C., Lee Y.J., Park H.J., Chong Y.H., Kang J.L. (2010). N-Acetylcysteine Inhibits RhoA and Promotes Apoptotic Cell Clearance during Intense Lung Inflammation. Am. J. Respir. Crit. Care Med..

[76] Anwar K.N., Fazal F., Malik A.B., Rahman A. (2004). RhoA/Rho-Associated Kinase Pathway Selectively Regulates Thrombin-Induced Intercellular Adhesion Molecule-1 Expression in Endothelial Cells via Activation of IκB Kinase β and Phosphorylation of RelA/P65. J. Immunol..

[77] Cammarano M.S., Minden A. (2001). Dbl and the Rho GTPases Activate NFκB by IκB Kinase (IKK)-Dependent and IKK-Independent Pathways. J. Biol. Chem..

[78] Montaner S., Perona R., Saniger L., Lacal J.C. (1999). Activation of Serum Response Factor by RhoA Is Mediated by the Nuclear Factor-ΚB and C/EBP Transcription Factors. J. Biol. Chem..

[79] Barroso L.C., Magalhaes G.S., Galvão I., Reis A.C., Souza D.G., Sousa L.P., Santos R.A.S., Campagnole-Santos M.J., Pinho V., Teixeira M.M. (2017). Angiotensin-(1-7) Promotes Resolution of Neutrophilic Inflammation in a Model of Antigen-Induced Arthritis in Mice. Front. Immunol..

[80] Vieira A.T., Galvão I., Macia L.M., Sernaglia É.M., Vinolo M.A.R., Garcia C.C., Tavares L.P., Amaral F.A., Sousa L.P., Martins F.S. (2017). Dietary Fiber and the Short-Chain Fatty Acid Acetate Promote Resolution of Neutrophilic Inflammation in a Model of Gout in Mice. J. Leukoc. Biol..

